# Developing Phototherapy-Based Interventions for Older Women: Insights From Therapists’ Experience

**DOI:** 10.1093/geroni/igaf122.1810

**Published:** 2025-12-31

**Authors:** Silvia Piol, Shoshi Keisari, Hod Orkibi

**Affiliations:** University of Haifa, Haifa, HaZafon, Israel; University of Haifa, Haifa, HaZafon, Israel; University of Haifa, Haifa, HaZafon, Israel

## Abstract

Phototherapy is a therapeutic approach in which trained therapists use photography and photographic materials to facilitate psychological growth and therapeutic change. This qualitative study explores how professionals employ phototherapy with older women, focusing on their perspectives on creative processes related to the aging female body. Aging women often navigate the impact of gendered ageism - the intersection of ageism and gender bias - which contributes to shape their self-perceptions and body image. In Western societies, where youthfulness is highly valued, older women may experience internalized ageist stereotypes that influence their sense of self. This study seeks to understand how phototherapy can serve as a means of reflection, validation, and self-expression in the face of these challenges. To investigate this, 16 international therapists working with middle-aged and older women through phototherapy were interviewed. Using reflexive thematic analysis, four key themes were generated: (1) encountering the aging body in photographs: navigating ageism and self-ageism; (2) exploring the aging body through visual metaphors and symbols; (3) photographs as a space for visibility and validation; and (4) claiming agency through active engagement in photography. The findings provide insights into how phototherapy techniques can be used to support aging women in exploring and expressing their embodied self-representations. These insights will inform the development of an arts-based intervention designed to address the psychosocial needs of older Italian women, fostering self-acceptance and agency through narrative and phototherapy practices.

